# Anti-fatigue activities of polysaccharides extracted from *Hericium erinaceus*

**DOI:** 10.3892/etm.2014.2139

**Published:** 2014-12-16

**Authors:** JIANQING LIU, CONGXIN DU, YIFEI WANG, ZHIHUA YU

**Affiliations:** 1 Section of Basketball, Wuhan Institute of Physical Education, Wuhan, Hubei 430079, P.R. China; 2 School of Physical Education, South-Central University for Nationalities, Hongshan, Wuhan, Hubei 430074, P.R. China

**Keywords:** forced swimming test, blood lactic acid, serum urea nitrogen, glycogen, superoxide dismutase, glutathione peroxidase, malondialdehyde, mice

## Abstract

*Hericium erinaceus* (HEP) is a notable medicinal fungus grown in China and other oriental countries. Polysaccharides from HEP have recently attracted considerable attention due to their numerous physiological activities. The objective of this study was to evaluate the anti-fatigue activity of HEP in a mouse model. After one week of acclimation, mice were randomly divided into four groups: a control group, a low-dose HEP-treated group, a moderate-dose HEP-treated group, and a high-dose HEP-treated group. The treated groups received HEP (50, 100 and 200 mg/kg, ig), while the control group received saline solution. Following treatment for 28 days, the mice performed a forced swimming test until they were exhausted, then the exhaustive swimming time was recorded along with certain biochemical parameters related to fatigue, including blood lactic acid (BLA), serum urea nitrogen (SUN), tissue glycogen, superoxide dismutase (SOD), glutathione peroxidase (GPx) and malondialdehyde (MDA). These results suggested that HEP has significant anti-fatigue activity by decreasing BLA, SUN and MDA content, as well as increasing tissue glycogen content and antioxidant enzyme activity. Based on these results, this study provided theoretical support for the application of HEP in the field of sports nutrition.

## Introduction

Fatigue may be defined as a situation in which the capacity for work is diminished and efficiency of accomplishment reduced (1), and it can be classified as physical or mental, depending on its cause. As examples, physical fatigue is caused by excessive exercise and mental fatigue is caused by sleep deprivation (2). Physical fatigue may be accompanied by deterioration in performance. Several factors have been identified to contribute to physical fatigue. First, exercise promotes consumption of energy sources including glycogen by mobilizing the internal energy metabolism to the maximum and using and depleting the energy source (3). Second, exercise causes the production and accumulation of metabolic products including lactic acid and ammonia in the body (4). Third, intense exercise produces a large quantity of reactive oxygen species (ROS) due to increased oxygen consumption. The superoxide anion radical (O_2_^−^) and hydrogen peroxide are generated as metabolic intermediates in the presence of oxygen. These may lead to a disturbance in the homeostasis of the endogenous antioxidative defense systems in the body, resulting in the development of fatigue (5). To date, pharmacological drugs or therapies used for treating fatigue have not been effective. Recently, interest has increased in the use of natural substance supplements for the attenuation of exercise-induced physical fatigue.

*Hericium erinaceus* (HEP) belongs to the Aphyllophorales, Hydnaecae and Hericium families and its fruiting body is called ‘Houtou’ in Chinese. It has been used as an edible and medicinal fungus in China and other oriental countries and areas for a number of years (6). The fungus contains essential constituents, including polysaccharides, lectins, proteins, lipids, hericenone, erinacol, erinacine and terpenoids (7). Polysaccharides from HEP have attracted considerable attention due to their numerous physiological activities, including immunomodulatory, hepatoprotective, antitumor, anti-aging, antioxidant and hypoglycemic activities (8–11). However, the anti-fatigue activity of HEP has not been investigated until now. The present study was designed to evaluate the anti-fatigue activity of HEP in a mouse model.

## Materials and methods

### Material

Dried fruiting bodies of HEP were obtained from a market in Wuhan city and identified by Professor Ming Wang from the Hubei Society for Microbiology (Wuhan, China). Voucher specimens (EH-SCN1391) were preserved in Hubei Natural Product Research Institute (Wuhan, China).

### Reagents and kits

Glucose was purchased from Guoyao Chemical Reagent Factory (Shanghai, China). The diagnostic kits for blood lactic acid (BLA), tissue glycogen and malondialdehyde (MDA) were purchased from Jiancheng Bioengineering Institute (Nanjing, China). The diagnostic kits for serum urea nitrogen (SUN) were purchased from Biosino Biotechnology and Science Inc. (Beijing, China). The diagnostic kits for superoxide dismutase (SOD) and glutathione peroxidase (GPx) were purchased from Comin Biotechnology Co., Ltd. (Suzhou, China). Other commercial chemicals used in the experiments were of analytical grade and were purchased from the Hongshan Reagent Company (Wuhan, China).

### Preparation of polysaccharides from HEP

Polysaccharides from HEP were prepared using the ethanol precipitation method as described by Li *et al* (12) and modified by Hui *et al* (13). Briefly, dried fruits of HEP were ground and extracted with petroleum ether at 60°C for 4 h to remove colored materials, oligosaccharides and small molecule materials under reflux in the apparatus. The organic solvent was separated by centrifugation (4390 × g, 20 min) and pretreated powder was obtained.

Next, the dried pretreated powder was extracted with boiling water (at a ratio of 1:30 w/v) for 4 h. The mixture was centrifuged (4390 × g, 20 min) and filtered, and the insoluble residue was treated again as mentioned above. The supernatant was incorporated and concentrated using a rotary evaporator at 50°C under a vacuum. The concentrated extract was precipitated by the addition of 95% (v/v) ethanol to a final concentration of 80% (v/v) and incubated for 12 h at 4°C. The precipitate was collected by centrifugation (4390 × g, 20 min) and then vacuum-dried at 40°C to afford crude polysaccharides from HEP. The polysaccharide content was measured by the phenol-sulfuric acid method using glucose as standard.

### Experiment animals

Male ICR mice, weighing 18–20 g at the beginning of the study, were purchased from Wanqian Jiaxing Biotechnology Co, Ltd. (Wuhan, China). They were fed under controlled environmental conditions of temperature (22±2°C) and a 12-h light/dark cycle, and maintained on a standard rodent diet and tap water *ad libitum* unless otherwise stated. All animals received professional humane care in compliance with the guidelines of the Ethical Committee of Wuhan Institute of Physical Education (Wuhan, China).

### Experiment design

After one week of acclimation, the mice were randomly divided into four groups (ten mice in each group) as follows: i) control (C) group: the mice were allowed free access to a standard rodent diet and treated with saline solution; ii) low-dose HEP-treated (LHT) group: the mice were allowed free access to a standard rodent diet and treated with 50 mg/kg bw of HEP; iii) moderate-dose HEP-treated (MHT) group: the mice were allowed free access to a standard rodent diet and treated with 100 mg/kg bw of HEP; iv) High-dose HEP-treated (HHT) group: the mice were allowed free access to a standard rodent diet and treated with 200 mg/kg bw of HEP.

HEP was dissolved in 2.0 ml saline solution, and the control group received the same volume of saline solution. Treatments were administered orally by gavage using a feeding needle, once a day for 28 consecutive days.

### Forced swimming test

One hour after the final treatment, forced swimming tests (FSTs) were conducted using the method described by Zhang *et al* (14). Tests were carried out in an acrylic plastic pool (90×45×45 cm) 35 cm deep with water maintained at 25±2°C. A tin wire (5% of body weight) was loaded on the tail root of each mouse. Exhaustion was determined by observing loss of coordinated movements and failure to return to the surface within 10 sec, and the exhaustive swimming time was immediately recorded.

### Analysis of biochemical parameters related to fatigue

After FSTs, the animals were sacrificed immediately by decapitation under anesthesia with sodium pentobarbital (40 mg/kg bw, ip). Blood samples of the mice were respectively collected in heparinized tubes and tubes without anticoagulant. Blood plasma was prepared by centrifugation at 4°C (2919 × g, 10 min) for the BLA analysis, and serum was prepared by centrifugation at 4°C (2919 × g, 15 min) for the SUN analysis. After the blood was collected, the gastrocnemius muscles and liver were rapidly excised and immediately frozen in liquid nitrogen and stored at −80°C for the tissue glycogen, SOD, GPx and MDA analysis.

### Analytical methods

The BLA content was determined based on the lactate dehydrogenase enzymatic method and the absorbance was read at 530 nm (15). SUN content was determined by the diacetyl monoxime colorimetric method and the absorbance was read at 520 nm (16). Glycogen content was determined by the sulfuric anthrone method and the absorbance was read at 620 nm (17). SOD activity was determined by the xanthine oxidase method (hydroxylamine method) and the absorbance was read at 550 nm (18). GPx activity was determined by the dithio-binitrobenzoic acid method and the absorbance was read at 412 nm (19). MDA content was determined by the thiobarbituric acid method and the absorbance was read at 532 nm (20).

### Statistical analysis

The results are expressed as the means ± standard deviation. Comparisons between groups were made using Student’s t-test, and P<0.05 was considered to indicate a statistically significant difference.

## Results and Discussion

### Effects of HEP on exhaustive swimming times

The FST, a behavioral test for rodents, previously used to predict the efficacy of antidepressants, has recently been used to examine whether certain agents have anti-fatigue activities (21). Prolonged swimming times in an FST indicate a decrease in fatigue (22).

**Figure 1:**
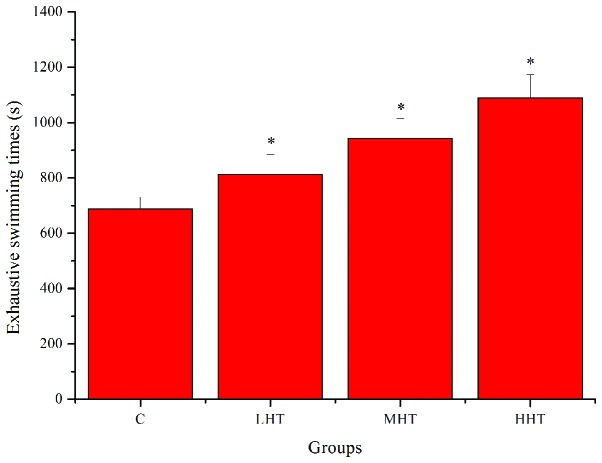
Effects of *Hericium erinaceus* on exhaustive swimming times. Values are expressed as the means ± SD. ^*^P<0.05, compared with the C group. C, control; LHT, low-dose HEP-treated group; MHT, moderate-dose HEP-treated group; HHT, high-dose HEP-treated group.

The effects of HEP on exhaustive swimming times are shown in Fig. 1. Exhaustive swimming times in the LHT, MHT and HHT groups were significantly longer (P<0.05) than that in the C group, by 18.15, 37.18 and 58.46%, respectively. These results indicated that HEP had significant anti-fatigue activity and was capable of elevating the exercise tolerance in mice.

### Effects of HEP on BLA and SUN content

In general, the swimming exercise is known to induce blood biochemical changes (23). The muscle produces a considerable amount of lactic acid when it obtains sufficient energy from anaerobic glycolysis, and the increased concentration of lactic acid brings about a reduction in the pH of muscle tissue and blood, which could induce various biochemical and physiological side effects, including glycolysis and phosphofructokinase and calciumion release, through muscular contraction (24). Therefore, BLA is a sensitive index of fatigue status. Urea is formed in the liver as the end product of protein metabolism. During digestion, protein is broken down into amino acids. Amino acids contain nitrogen, which is removed as NH_4_^+^ (an ammonium ion), while the remainder of the molecule is used to produce energy and other substances required by the cell (14). There is a positive correlation between the urea nitrogen *in vivo* and exercise tolerance. In other words, the worse the body is adapted for exercise tolerance, the more significantly the urea nitrogen level increases (25). Thus, SUN is another sensitive index of fatigue status.

**Figure 2:**
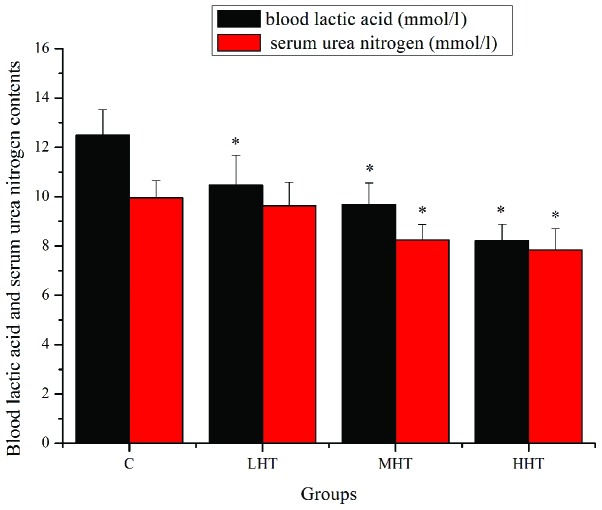
Effects of *Hericium erinaceus* on blood lactic acid and serum urea nitrogen content. Values are expressed as the means ± SD. ^*^P<0.05, compared with the C group. C, control; LHT, low-dose HEP-treated group; MHT, moderate-dose HEP-treated group; HHT, high-dose HEP-treated group.

The effects of HEP on BLA and SUN content are shown in Fig. 2. The BLA content of the LHT, MHT and HHT groups was significantly lower (P<0.05) than that of the C group, by 16.43, 28.90 and 52.13%, respectively. The SUN content of the MHT and HHT groups was significantly lower (P<0.05) than that of the C group, by 20.87 and 27.04%, respectively. The SUN content of the LHT group was also lower, but not significantly (P>0.05). These results indicated that HEP effectively delayed the increase in BLA, reduced the catabolism of protein for energy and increased the adaptive capacity to exercise load, which ultimately postponed the appearance of physical fatigue.

### Effects of HEP on glycogen content in liver and muscle

Energy for exercise is derived initially from the breakdown of glycogen in muscle. Following strenuous exercise, it may be depleted, and at later stages the energy will be derived from hepatic glycogen (26). Therefore, the depletion of glycogen stores may be a significant factor in the development of fatigue.

**Figure 3:**
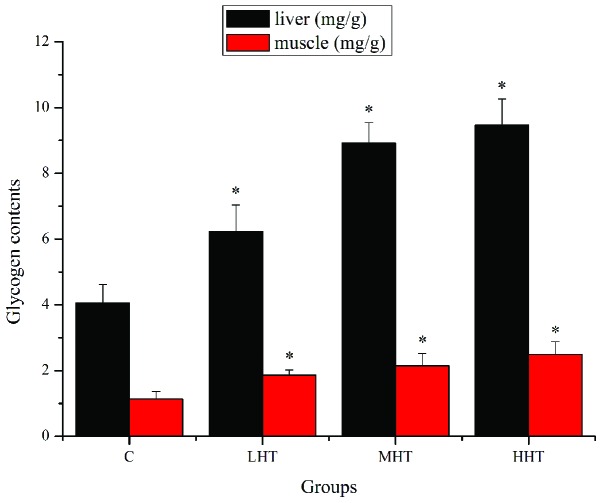
Effects of *Hericium erinaceus* on glycogen content in liver and muscle. Values are expressed as the means ± SD. ^*^P<0.05, compared with the C group. C, control; LHT, low-dose HEP-treated group; MHT, moderate-dose HEP-treated group; HHT, high-dose HEP-treated group.

The effects of HEP on glycogen content in liver and muscle are shown in Fig. 3. The liver glycogen content of the LHT, MHT and HHT groups was significantly higher (P<0.05) than that of the C group, by 53.45, 119.70 and 133.25%, respectively. The muscle glycogen content of the LHT, MHT and HHT groups was significantly higher (P<0.05) than that of the C group, by 64.60, 90.27 and 120.35%, respectively. These results indicated that HEP may contribute to the improvement of metabolic control of exercise and the activation of energy metabolism (27), which could ameliorate physical fatigue by increasing the storage of glycogen in liver and muscle.

### Effects of HEP on SOD and GPx activity in liver and muscle

Previous studies have reported that ROS are responsible for exercise-induced protein oxidation, and contribute significantly to muscle fatigue (28). Two major classes of endogenous protective mechanisms, enzymatic and non-enzymatic antioxidants, work to reduce the harmful effects of ROS in cells (29). SOD and GPx constitute the principal components of the enzymatic antioxidant defense systems. There is growing evidence indicating that the improvement in the activity of SOD and GPx help fight against fatigue and protect cells from oxidative damage (30,31).

**Figure 4:**
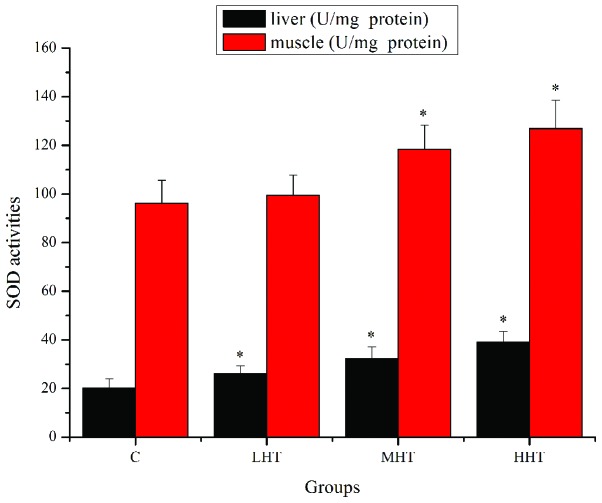
Effects of *Hericium erinaceus* on superoxide dismutase (SOD) activity in liver and muscle. Values are expressed as the means ± SD. ^*^P<0.05, compared with the C group. C, control; LHT, low-dose HEP-treated group; MHT, moderate-dose HEP-treated group; HHT, high-dose HEP-treated group.

**Figure 5:**
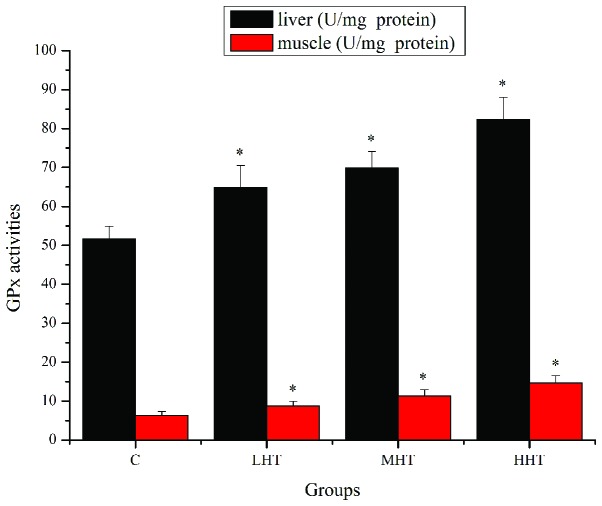
Effects of *Hericium erinaceus* on glutathione peroxidase (GPx) activity in liver and muscle. Values are expressed as the means ± SD. ^*^P<0.05, compared with the C group. C, control; LHT, low-dose HEP-treated group; MHT, moderate-dose HEP-treated group; HHT, high-dose HEP-treated group.

The effects of HEP on SOD activity are shown in Fig. 4. The SOD activity in the liver of the LHT, MHT and HHT groups was significantly higher (P<0.05) than in that of the C group, by 29.96, 60.66 and 94.39%, respectively. SOD activity in the muscle of the MHT and HHT group was significantly higher (P<0.05) than in that of the C group, by 22.96 and 31.81%, respectively. SOD activity in the muscle of the LHT group was also higher but not significantly (P>0.05). The effects of HEP on GPx activity are shown in Fig. 5. GPx activity in the liver of the LHT, MHT and HHT groups was significantly higher (P<0.05) than in that of the C group, by 25.50, 35.11 and 59.39%, respectively. GPx activity in the muscle of the LHT, MHT and HHT groups was significantly higher (P<0.05) than in that of the C group, by 37.74, 78.77 and 130.35%, respectively. These results indicated that HEP was able to upregulate antioxidant enzymes activity to ameliorate physical fatigue.

### Effects of HEP on MDA content in muscle and liver

It is generally accepted that fatigue causes the release of ROS, which leads to lipid peroxidation of the membrane structure and causes oxidative damage to cellular macromolecules (32). MDA is the breakdown product of the major chain reactions leading to the oxidation of polyunsaturated fatty acids and thus serves as an indicator of lipid peroxidation (33). A number of studies have reported that exhaustive exercise increased the MDA content in liver and muscle tissues in rats and mice (34,35).

**Figure 6:**
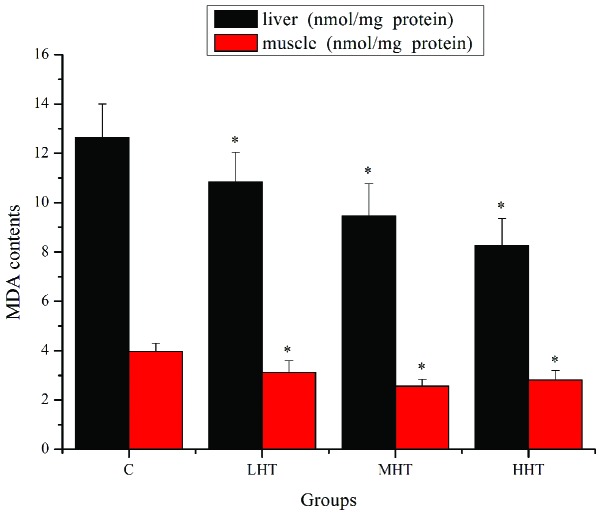
Effects of *Hericium erinaceus* on malondialdehyde (MDA) content in muscle and liver. Values are expressed as means ± SD. ^*^P<0.05, compared with the C group. C, control; LHT, low-dose HEP-treated group; MHT, moderate-dose HEP-treated group; HHT, high-dose HEP-treated group.

The effects of HEP on MDA content are shown in Fig. 6. The MDA content in the liver of the LHT, MHT and HHT groups was significantly lower (P<0.05) than in that of the C group, by 16.71, 33.62 and 52.84%, respectively. The MDA content in the muscle of the LHT, MHT and HHT groups was significantly lower (P<0.05) than that of the C group, by 26.92, 54.69 and 40.93%, respectively. These results indicated that HEP reduced lipid peroxidation and prevented exercise-induced oxidative damage.

The results of the present study suggest that HEP possesses significant anti-fatigue activity by decreasing BLA, SUN and MDA content, and increasing tissue glycogen content and antioxidant enzyme activity. Based on these results, this study provides theoretical support for the application of HEP in the field of sports nutrition.
